# Suppression of obesity by melatonin through increasing energy expenditure and accelerating lipolysis in mice fed a high-fat diet

**DOI:** 10.1038/s41387-022-00222-2

**Published:** 2022-10-07

**Authors:** Liang Xu, Dandan Li, Haoran Li, Ouyang Zhang, Yaxin Huang, Hengrong Shao, Yajiao Wang, Suili Cai, Yuqin Zhu, Shengnan Jin, Chunming Ding

**Affiliations:** 1 School of Laboratory Medicine and Life Sciences, Wenzhou Medical University, Wenzhou, 325035 Zhejiang China; 2 Key Laboratory of Laboratory Medicine, Ministry of Education, Wenzhou Medical University, Wenzhou, 325035 Zhejiang China

**Keywords:** Obesity, Metabolic syndrome

## Abstract

**Backgrounds/objectives:**

Melatonin promotes brown adipose tissue (BAT) activity, leading to body mass reduction and energy expenditure. However, the mechanisms governing these beneficial effects are not well-established. This study aimed to assess the effects of (1) melatonin on BAT and energy metabolism, and (2) fibroblast growth factor 21 (FGF21) in BAT-mediated thermogenesis.

**Methods:**

Male C57BL/6 J mice received a high-fat diet (HFD) or normal chow, accompanied by intraperitoneal injection of 20 mg/kg melatonin for 12 weeks. FGF21^−/−^ mice consumed an HFD with or without melatonin for 8 weeks.

**Results:**

Melatonin attenuated weight gain, insulin resistance, adipocyte hypertrophy, inflammation, and hepatic steatosis induced by the HFD and increased energy expenditure. Furthermore, melatonin improved cold tolerance by increasing BAT uncoupling protein 1 (UCP1) expression and producing heat. Notably, melatonin resulted in a shift in energy metabolism favouring the utilization of fat, and it increased FGF21 in circulating and metabolic tissues and skeletal muscle phosphorylation of AMP-activated protein kinase. However, melatonin did not protect against obesity, insulin resistance, and energy expenditure in HFD-fed FGF21^−/−^ mice.

**Conclusions:**

Melatonin suppressed obesity and insulin resistance resulting from the HFD by enhancing BAT activity and energy expenditure, and these effects were dependent on FGF21.

## Introduction

Obesity occurs when energy balance is altered and energy intake surpasses expenditure, the latter of which is influenced by basal metabolic rate, physical activity, and thermogenesis [1]. Excess energy from nutrients is stored primarily in white adipose tissue (WAT), leading to higher total body mass. Brown adipose tissue (BAT) regulates energy homeostasis and transforms fat into heat. Uncoupling protein 1 (UCP1) catalyzes to mitochondrial proton conductance and protection against hypothermia and obesity [2]. Notably, promoting the function of classic brown fat or the browning of WAT (known as brown-like adipocytes or beige adipocytes) help protect against obesity and its metabolic complications in animal studies [3, 4]. Therefore, promoting BAT and/or beige WAT function has potential as a therapeutic approach to mitigate the current obesity epidemic.

The hormone melatonin is secreted nocturnally by the pineal gland, regulates circadian rhythms, and functions as an antioxidant. In addition, melatonin regulates energy metabolism by limiting obesity [5–8] independent of food intake [9, 10]. However, it exerts inconsistent effects on locomotor activity [11]. These observations suggest that melatonin may have thermogenic actions in the body. Melatonin leads to increased BAT, which in turn leads to metabolic improvements including reduced body mass and increased energy expenditure [12–14]. In addition, melatonin promotes WAT browning and thermogenic effects in Zücker diabetic fatty rats [15]. Removing the pineal gland eliminates melatonin, which abolishes the BAT response to metabolic challenges in humans [14]. However, replacing melatonin reverses this effect, and increases the expression of thermogenic genes [14], which suggests that melatonin is an important component of the BAT activation pathway. However, the mechanisms by which melatonin promotes BAT activity and WAT browning warrant further examination.

Fibroblast growth factor 21 (FGF21) regulates the differentiation to brown adipocytes [16, 17]. FGF21 upregulates UCP1 and peroxisome proliferator-activated receptor γ coactivator (PGC)-1α following exposure to cold, thus promoting thermogenesis in adipose tissue [18]. Therefore, this study explored the mechanism of melatonin regulating BAT function and whether it depends on FGF21.

## Materials and methods

### Animals and treatment

Eight-week-old C57BL/6 male mice (China National Laboratory, Shanghai, China) were randomized to four groups for 12 weeks: (1) normal chow with vehicle (NC + Veh, *n* = 8; 10% kcal from fat) (Supplementary Table 1); (2) NC with 20 mg/kg melatonin (NC + MEL, *n* = 7); (3) high-fat diet with vehicle (HFD + Veh, *n* = 7; 60% kcal from fat; #D12492; Research Diets, New Brunswick, NJ, USA, Supplementary Table 1); and (4) HFD with 20 mg/kg melatonin (HFD + MEL, *n* = 6). The HFD and HFD + MEL mice were pair-fed.

Eight-week-old male FGF21^−/−^ mice (C57BL/6; Cyagen Biosciences Inc., Suzhou, China) were pair-fed HFD or HFD + MEL (20 mg/kg) groups for 8 weeks. After feeding in the above-described manner, all mice were fasted overnight, and then were anesthetized and sacrificed by pentobarbital sodium (50 mg/kg) injected intraperitoneally (i.p.) at 9:00 am.

The melatonin dose was selected based on previous mouse studies [19, 20]. Melatonin was used as described previously [21]. Briefly, a stock melatonin solution (0.5% v/v) was prepared with melatonin (#M5250; Sigma-Aldrich Corporation, St. Louis, MO, USA) dissolved in anhydrous alcohol. Before dark, mice received i.p. injection of 200 μL solution with melatonin (20 mg/kg) in phosphate-buffered saline (PBS) for 12 weeks; parallel-group animals were administered a vehicle comprised of 0.5% ethanol in PBS.

Mice were housed with standard temperature (22 ± 2 °C), humidity (45 ± 5%), and a 12 h light cycle. The Wenzhou Medical University Animal Experiment Committee approved the protocol used in this study and it was performed according to institutional guidelines.

### Indirect calorimetry

Following 3 weeks of melatonin treatment, mice were placed in individual calorimetry chambers (Oxymax; Columbus Instruments, Columbus, OH, USA) to determine energy metabolism before body weight differences appeared. Daily food intake, body weight, and calorimetry were recorded during the 3-day acclimation and 2-day experimental periods. For the experimental period, oxygen consumed (VO_2_) and carbon dioxide produced (VCO_2_) were assessed, and the respiratory exchange ratio (RER, VCO_2_ / VO_2_), and energy expenditure (VO_2_ × [3.815 + (1.232 × RER)]) were determined.

### Body composition measurement

Body composition was measured using MRI equipment (EchoMRI) before sacrifice.

### Histology and immunohistochemistry

Paraffin-embedded BAT, epididymal WAT (eWAT), liver, and skeletal muscle were sectioned and stained with hematoxylin and eosin (H&E). Immunohistochemistry was performed on the liver and eWAT sections using F4/80, and on the BAT sections with UCP1 [22].

### Glucose, insulin, and lipid measurements

Plasma glucose, insulin, FGF21, triglycerides (TG), hepatic TG, total cholesterol (TC), non-esterified fatty acid (NEFA), and alanine aminotransferase (ALT) were determined as previously described [23, 24].

### Glucose and insulin tolerance tests

Mice underwent a glucose tolerance test (GTT) following 12 weeks of feeding, for which they received an i.p. injection of glucose (2 g/kg) after fasting overnight. One week later, they were subjected to an insulin tolerance test (ITT). After fasting for 4 h, they received i.p. injections of human insulin (0.5 U/kg). Blood glucose was measured using a glucometer (Roche, Manheim, Germany) before injection and at 30-min intervals for 2 h following the injection.

### Insulin signaling in vivo

Ten minutes before sacrifice, the mice (three mice per group) were given i.p. injections of insulin (10 U/kg) to detect Akt (Ser473) phosphorylation in vivo.

### Quantitative RT-PCR analyses

TRIzol^TM^ (Invitrogen, Thermo Fisher, Carlsbad, CA, USA) was used for total RNA isolation and concentrations were determined using spectrophotometry (NanoDrop 5000, Thermo Fisher, Waltham, MA, USA). cDNA synthesis was performed with a high-capacity reverse transcription kit (Applied Biosystems, Foster City, CA, USA). mRNA expression was measured by qPCR with SYBR Green [25]. Primers are listed in Supplementary Table 2.

### Immunoblotting analyses

Mice tissues were lysed using RIPA buffer (EMD Millipore, Billerica, MA, USA) containing phosphatase and protease inhibitors (Sigma-Aldrich) and protein concentrations were measured using a BCA assay Kit (Pierce, Bonn, Germany). The lysates were incubated at 4 °C overnight with primary antibodies (Supplementary Table 3), followed by secondary antibodies (Cell Signaling Technology, Danvers, MA, USA). Proteins were detected by chemiluminescence (Bio-Rad, Hercules, CA, USA) and visualized using an imaging system (ImageLab, ver. 3.0; Bio-Rad).

### Statistical analyses

Data are presented as mean ± standard error with significance at *P* < 0.05. Two-tailed student’s *t*-test was used to compare means between two groups, while one-way analysis of variance (ANOVA) was employed for more than two groups. When the latter indicated significant difference, individual group differences were computed using Bonferroni test. For energy expenditure, ANCOVA analysis was performed with body weight as a covariate [26].

## Results

### Melatonin attenuates HFD-induced weight gain and adiposity in mice

**Fig. 1:**
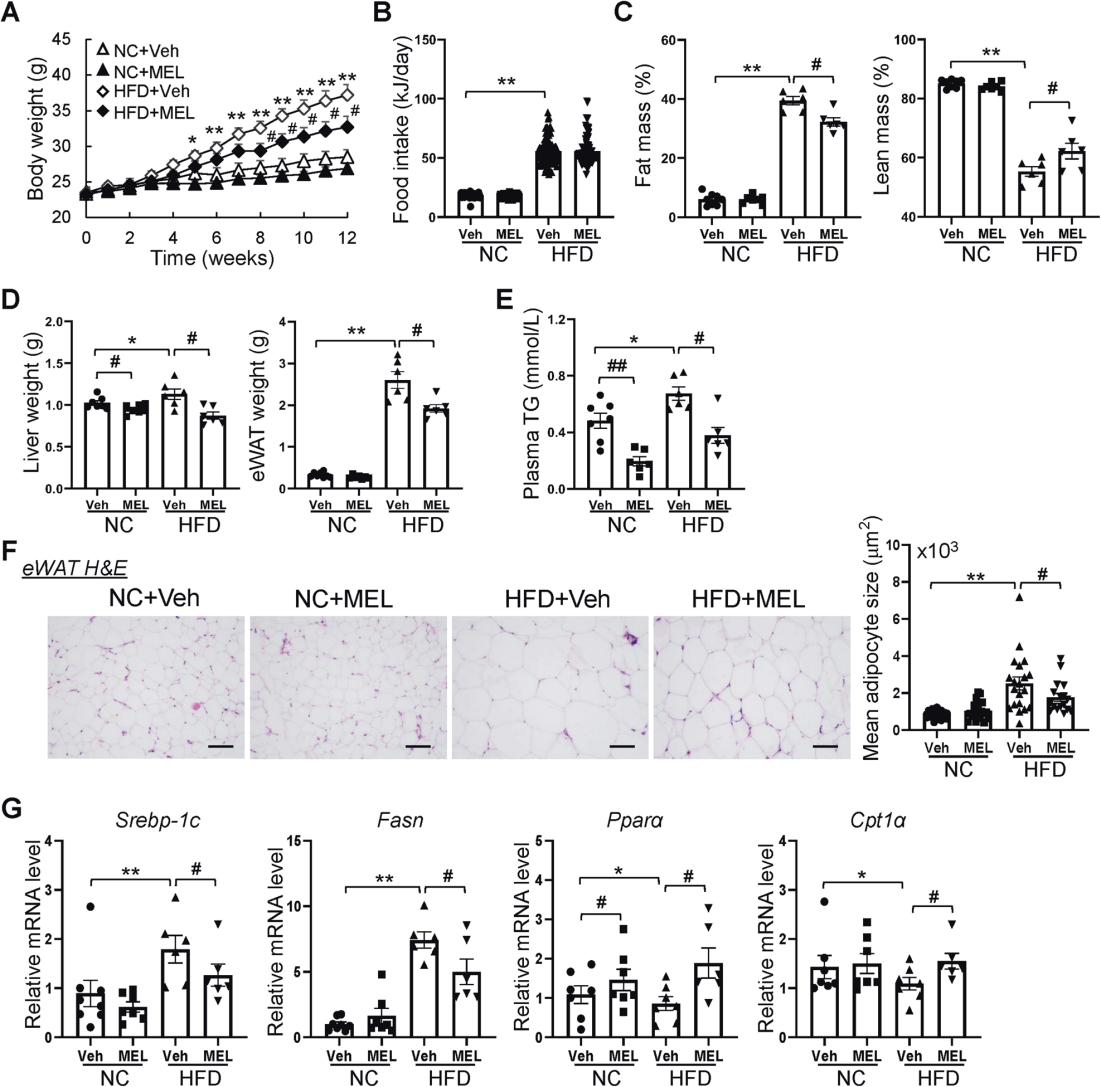
Melatonin decreases body weight gain and adipocyte hypertrophy in HFD-fed mice. **A** Body weight of mice. **B** Food intake of mice. **C** Fat mass and lean mass of mice. **D** Liver and eWAT weight of mice. **E** Plasma triglyceride (TG) content. **F** H&E-stained eWAT sections. Scale bars = 100 μm. **G** mRNA levels of lipogenesis-related and fatty acid oxidation-related genes in eWAT. Data are mean ± SEM, *n* = 5–8. Significance was determined by one-way ANOVA. *p < 0.05, **p < 0.01 vs. NC + Veh mice; ^*#*^*p* < 0.05, ^##^*p* < 0.01, vs. HFD + Veh mice.

To assess the metabolic effects of melatonin, we intraperitoneally injected the mice daily with melatonin (20 mg/kg) or vehicle (Veh) for 12 weeks, following a previous study [20]. The NC + MEL treatment did not significantly affect body weight (Fig. 1A), food intake (Fig. 1B), fat mass (Fig. 1C), or lean mass (Fig. 1C), which suggests that melatonin does not influence growth under conditions of energy balance. In contrast, melatonin attenuated weight gain in the HFD-fed mice compared to NC (Fig. 1A). This was due to the reduced fat mass, although the lean mass ratio increased (Fig. 1C). Moreover, melatonin reduced the weights of the liver and eWAT in HFD-induced obese (DIO) mice (Fig. 1D). Accordingly, the plasma TG level also decreased in response to the melatonin treatment in HFD and NC mice (Fig. 1E). Along with increased adiposity, histological evaluation of eWAT revealed larger adipocytes in HFD mice than NC mice (Fig. 1F). Notably, melatonin treatment resulted in reduced adipocyte size in the HFD mice (Fig. 1F). Additionally, expression of genes related to synthesis of fatty acids (*Srebp-1c* and *fasn)* decreased consistently in response to melatonin (Fig. 1G). In contrast, expression of genes related to β-oxidation of fatty acids (*Pparα* and *Cpt-1α*) were higher in melatonin-treated mice (Fig. 1G).

### Melatonin prevents reduced energy expenditure in HFD mice

**Fig. 2:**
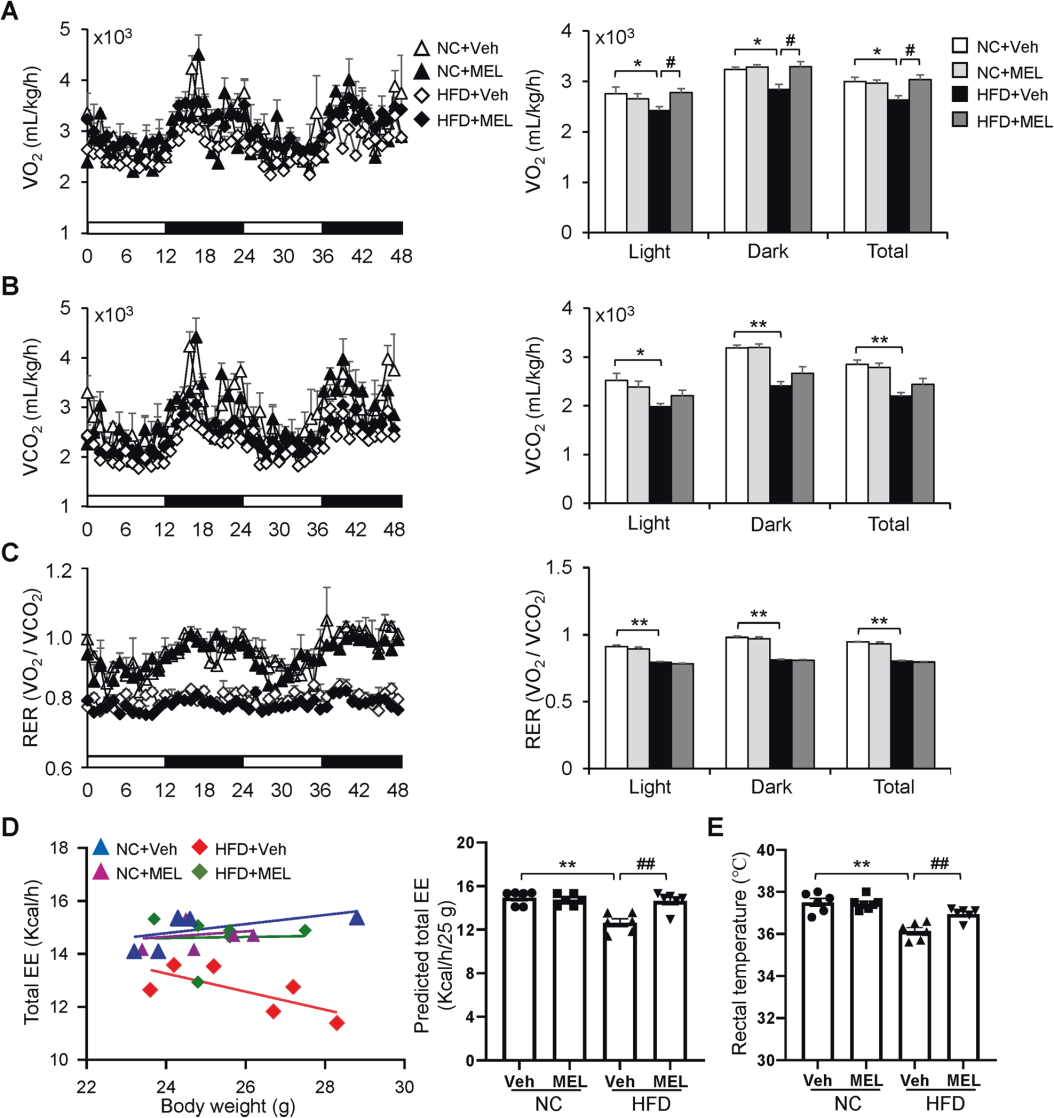
Melatonin increases energy expenditure in DIO mice. **A** VO_2_ of the mice. **B** VCO_2_ of the mice. **C** Respiratory exchange ratio (RER) of the mice. **D** Energy expenditure (EE) plotted against body weight and ANCOVA predicted energy expenditure at a given body weight of 25 g in mice. The ANCOVA bodyweight effect is *p* = 0.003. **E** Rectal temperature of the mice. Data are mean ± SEM, *n* = 5–8. Significance was determined by one-way ANOVA. *p < 0.05, **p < 0.01 vs. NC + Veh mice; ^*#*^*p* < 0.05, ^*##*^*p* < 0.01, vs. HFD + Veh mice.

To evaluate the effects of melatonin on energy balance, we measured metabolic parameters in melatonin-treated mice using indirect calorimetry. As anticipated, HFD mice had lower VO_2_ and VCO_2_ compared to NC mice (Fig. 2A, B). Strikingly, melatonin-treated HFD mice had higher oxygen consumption and carbon dioxide production than HFD controls, indicating an increase in energy expenditure (Fig. 2A, B, D), while RER was similar for the two groups (Fig. 2C). This suggests that under HFD conditions, melatonin promoted the utilization of glucose and lipid. Melatonin also induced a ~1 °C increase in core temperature among HFD mice (Fig. 2E). In contrast, melatonin did not affect the VO_2_ or VCO_2_ of NC mice (Fig. 2A, B). This suggests that melatonin acts to reduce obesity by increasing energy expenditure.

### Melatonin promotes brown adipocyte function in DIO and cold-exposed mice

**Fig. 3:**
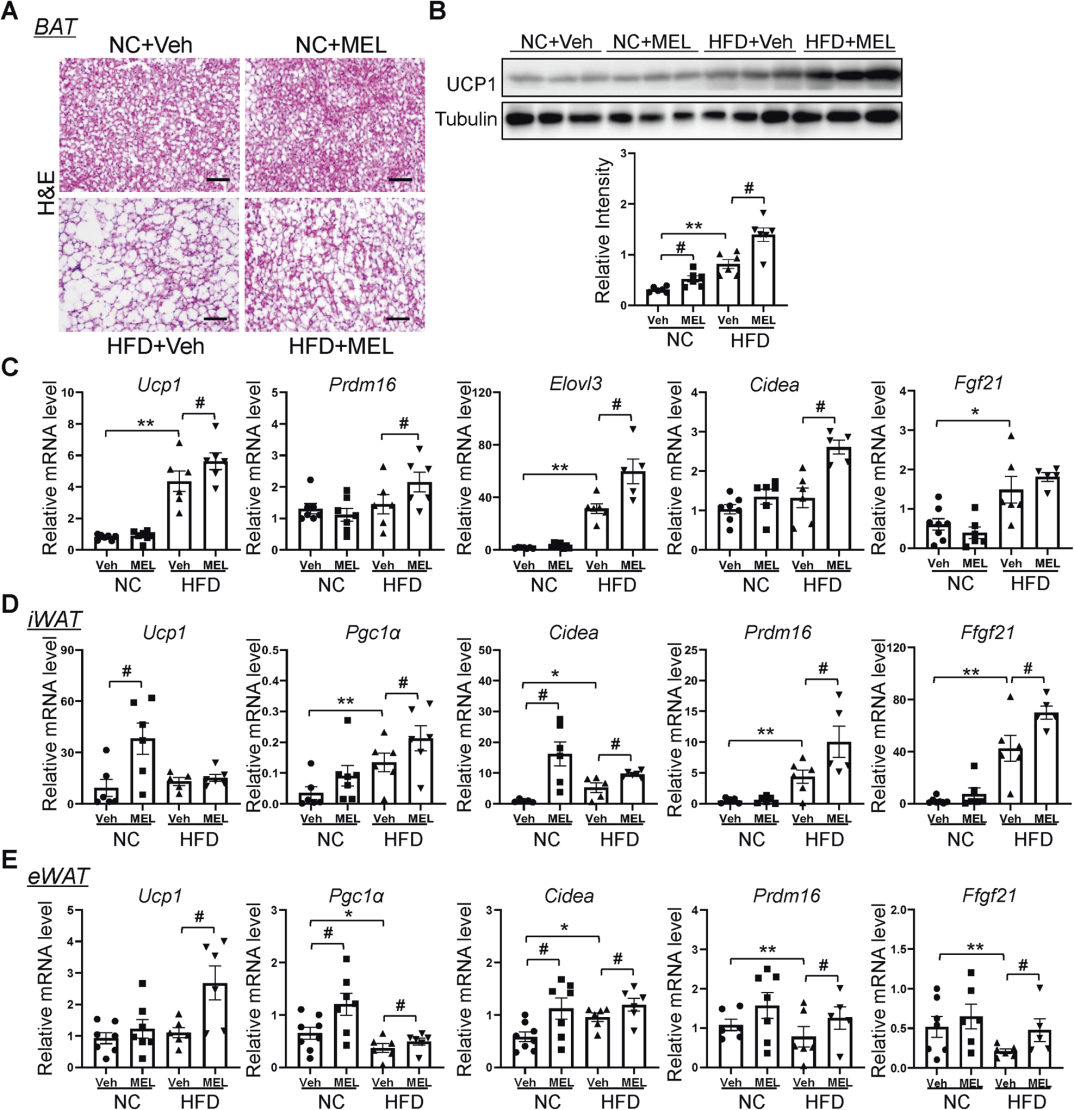
Melatonin increases UCP1 expression in BAT and WAT of HFD-stimulated mice. **A** H&E-stained BAT sections of NC-fed and HFD-fed mice. Scale bars = 100 μm. **B** Immunoblots of UCP1 in BAT of NC-fed and HFD-fed mice. **C** mRNA expression of thermogenesis-related genes in BAT of NC-fed and HFD-fed mice. **D**, **E** mRNA expression of beige-related genes in iWAT (**D**) and eWAT (**E**) of NC-fed and HFD-fed mice. Data are mean ± SEM, *n* = 5–8. Significance was determined by one-way ANOVA. *p < 0.05, **p < 0.01 vs. NC + Veh mice; ^#^*p* < 0.05, ^##^*p* < 0.01, vs. HFD + Veh mice.

The elevated body temperature and energy expenditure of melatonin-treated HFD mice may indicate increased adaptive thermogenesis. Melatonin led to reduced lipid accumulation and increased BAT UCP1 in HFD mice (Fig. 3A, B). It also resulted in upregulated expression of thermogenic genes (*Ucp1*, *Prdm16*, *Elovl3*, and *Cidea*) in DIO mice (Fig. 3C). UCP1-expressing brown-like adipocytes are found in WAT and play a regulatory role in thermogenesis [4]. Here, melatonin increased beige-selective gene expression in eWAT and inguinal WAT (iWAT) (*Ucp1*, *Cidea*, *Pgc1α*, *Prdm16*, and *Fgf21*) (Fig. 3D, E).

**Fig. 4:**
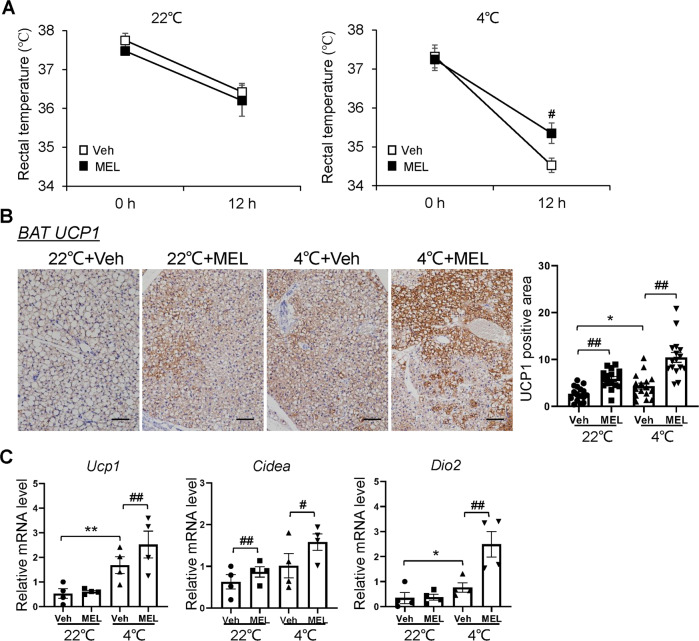
Melatonin increases adaptive thermogenesis of cold-stimulated mice. **A** Rectal temperature of 22 °C- and 4 °C-stimulated mice. **B** UCP1-stained BAT section of 22 °C- and 4 °C-stimulated mice. **C** mRNA expression of thermogenesis-related genes in BAT of mice. Data are mean ± SEM, *n* = 4. Significance was determined by one-way ANOVA. *p < 0.05, **p < 0.01 vs. 22 °C + Veh mice; ^#^*p* < 0.05, ^*##*^*p* < 0.01, vs. 4 °C + Veh mice.

To further assess whether melatonin results in altered whole-body thermogenic capacity, the NC-fed mice (8-week-old, *n* = 4) were adaptively treated with melatonin for 1 week, and then the mice were transferred from a normal condition (22 °C) to cold environment (4 °C) before turning off the light and kept for 12 h. The basal rectal temperatures were similar among the four groups of mice (22 °C + Veh = 37.8 °C ± 0.2, 22 °C + MEL = 37.5 °C ± 0.1, 4 °C + Veh = 37.3 °C ± 0.3, and 4 °C + MEL = 37.3 °C ± 0.3) (Fig. 4A). However, after transferring the mice to the 4 °C environment, melatonin protected the mice against the cold challenge by increasing body temperature (4 °C + Veh = 34.5 °C ± 0.2, 4 °C + MEL = 35.4 °C ± 0.3, *p* < *0.05*) (Fig. 4A). Thus, the melatonin-treated mice had better cold tolerance. Accordingly, MEL increased the amount of UCP1^+^ cells in BAT among cold-exposed mice (Fig. 4B) along with the expression of thermogenic genes (*Ucp1*, *Cidea*, and *Dio2*) (Fig. 4C). Taken together, melatonin appeared to promote BAT function and fat browning in obese mice, leading to increased energy expenditure.

### Melatonin enhances skeletal muscle fatty acid oxidation in obese mice

**Fig. 5:**
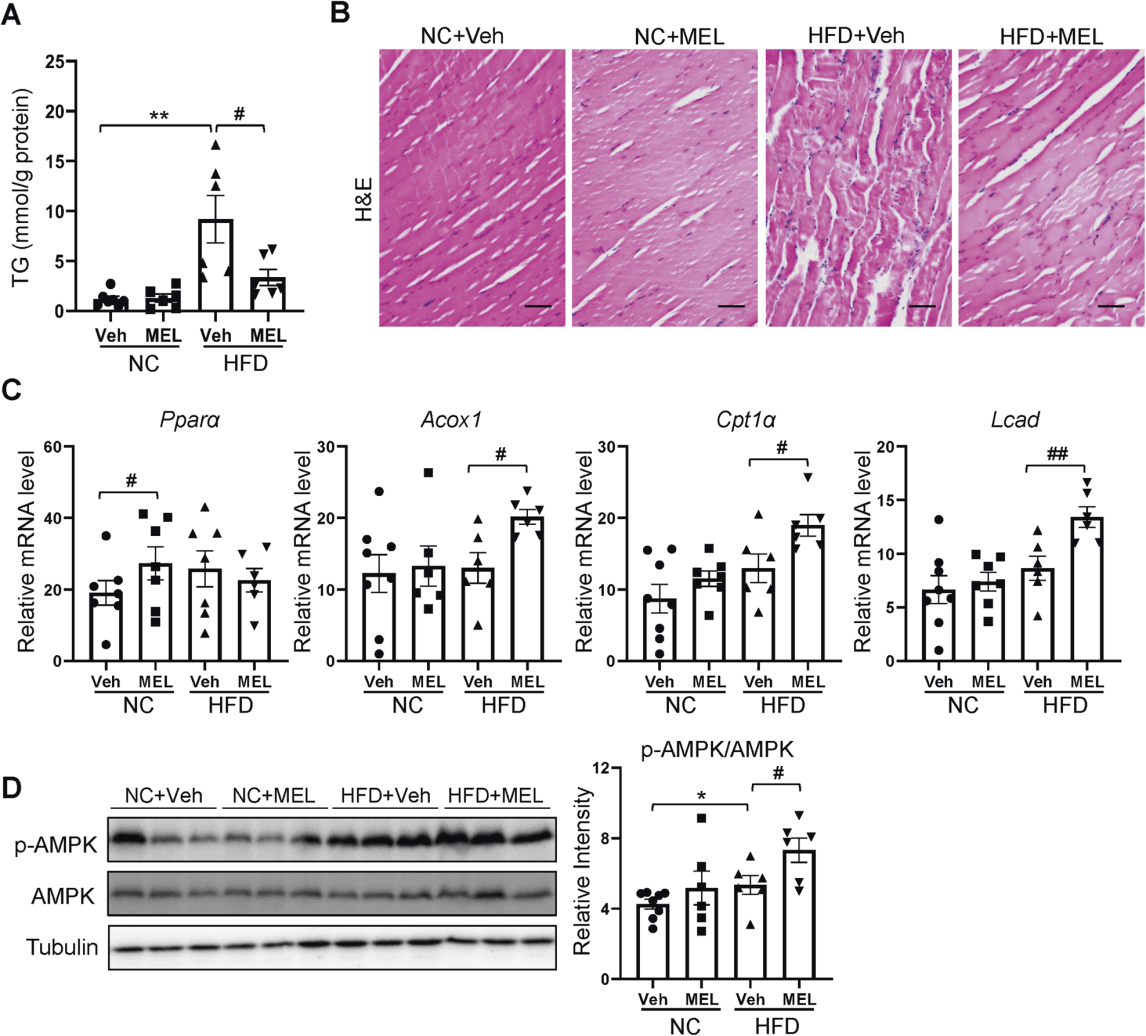
Melatonin increases fatty acid oxidation in skeletal muscle. **A** Muscle TG content. **B** H&E-stained skeletal muscle section. Scale bars = 100 μm. **C** mRNA expression of fatty acid oxidation-related genes in skeletal muscle. **D** Immunoblots of phosphorylated Th172 AMPK and AMPK in skeletal muscle. Data are mean ± SEM, *n* = 5–8. Significance was determined by one-way ANOVA. *p < 0.05, **p < 0.01 vs. NC + Veh mice; ^*#*^*p* < 0.05, ^##^*p* < 0.01, vs. HFD + Veh mice.

In addition to brown fat, skeletal muscle is another major energy-consuming organ, regulating fatty acid use in rodents [27]. Melatonin suppressed HFD-induced accumulation of TG in skeletal muscle (Fig. 5A). Accordingly, the skeletal muscle of the HFD mice exhibited an irregular fiber structure and excess ectopic fat deposition, whereas the melatonin treatment restored the abnormal muscle myofibers and fat accumulation, similar to that of NC-fed mice (Fig. 5B). These reductions in muscle fat accumulation by melatonin were associated with upregulated expression of genes related to β-oxidation of fatty acids (*Pparα*, *Acox1, Cpt1α*, and *Lcad*) (Fig. 5C). Furthermore, AMP-activated protein kinase (AMPK) α (Thr172) phosphorylation increased in response to melatonin (Fig. 5D), which suggests that melatonin activates AMPK and promotes skeletal muscle fat oxidation.

### Melatonin ameliorates insulin resistance and hepatic steatosis induced by HFD

**Fig. 6:**
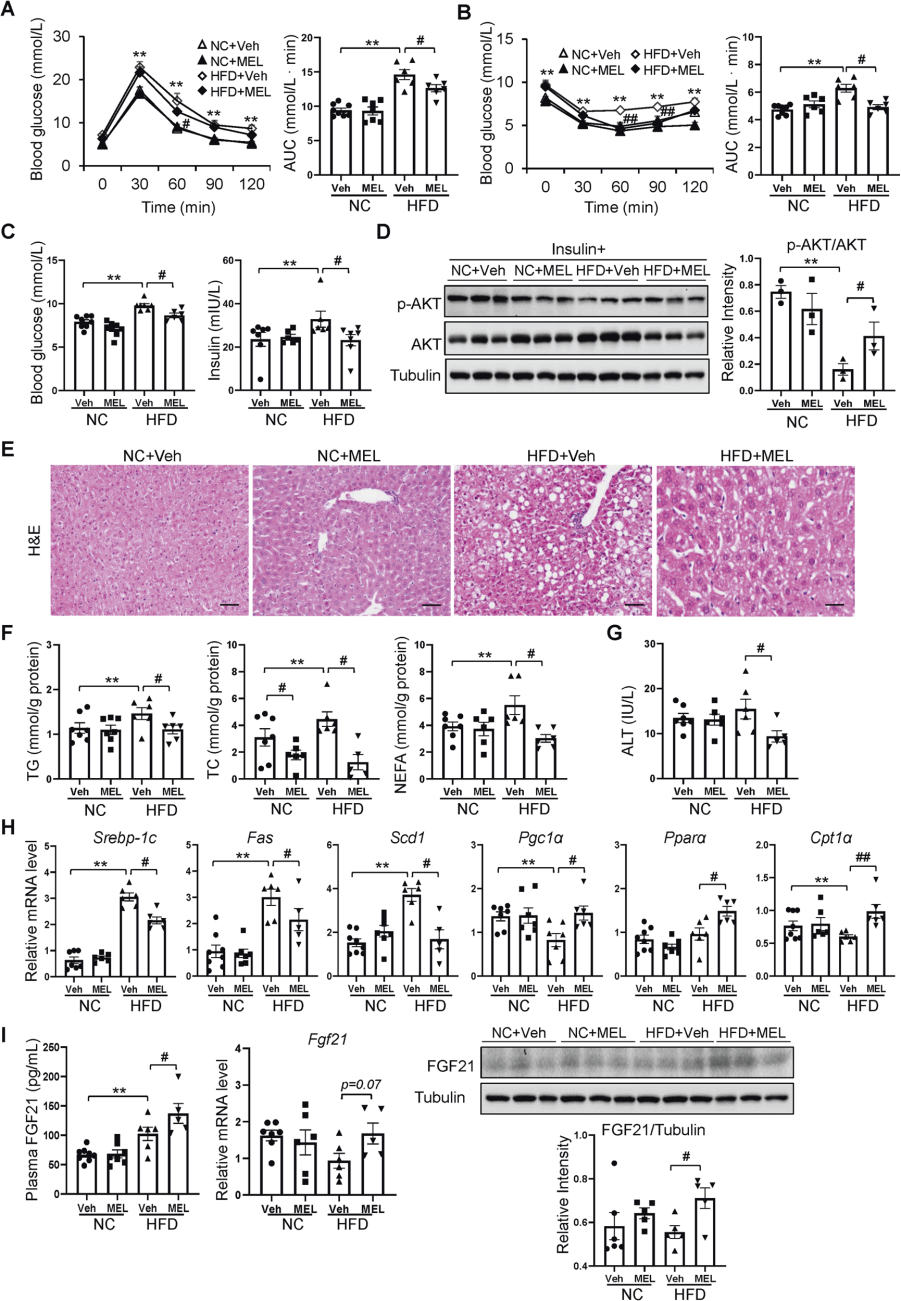
Melatonin ameliorates HFD-induced insulin resistance and hepatic steatosis. **A** Glucose tolerance test. **B** Insulin tolerance test. **C** Blood glucose and plasma insulin levels. **D** Immunoblots of phosphorylated Ser473 Akt (p-Akt) and Akt in the liver. **E** H&E-stained liver sections. Scale bars = 100 μm. **F** Hepatic lipid levels. **G** Plasma ALT content. **H** mRNA levels of lipogenesis-related and fatty acid oxidation-related genes in the liver. **I** The levels of FGF21 in plasma and liver of mice. Data are mean ± SEM, *n* = 5–8. Significance was determined by one-way ANOVA. *p < 0.05, **p < 0.01 vs. NC + Veh mice; ^#^*p* < 0.05, ^*##*^*p* < 0.01, vs. HFD + Veh mice. AUC area under curve, TG triglyceride, TC total cholesterol, NEFA non-esterified fatty acid, ALT alanine aminotransferase.

The glucose tolerance test revealed that melatonin markedly ameliorated glucose metabolism (Fig. 6A) and improved insulin sensitivity in HFD mice (Fig. 6B). Administering melatonin also normalized the hyperglycosemia and hyperinsulinemia seen in DIO mice (Fig. 6C). Accordingly, Akt (Ser473) phosphorylation was elevated in liver tissue of HFD + MEL mice (Fig. 6D).

The HFD led to inflammation, hepatic steatosis, and eventually fatty liver disease. Administering melatonin attenuated hepatic steatosis induced by the HFD (Fig. 6E) and led to additional reductions in liver TG, TC, and NEFA contents (Fig. 6F). In addition, melatonin alleviated HFD-induced liver dysfunction according to the plasma ALT level (Fig. 6G)., and this was associated with downregulation of lipogenic genes (*Fas*, *Scd1*, and *Srebp-1c*,) and increased expression of genes related to mitochondrial β-oxidation of fatty acids (*Pgc-1α*, *Pparα*, and *Cpt-1α*) (Fig. 6H). Furthermore, levels of FGF21 in plasma and liver were increased in response to melatonin in DIO mice (Fig. 6I). This indicates that FGF21 alters energy metabolism favoring fat utilization in response to the melatonin treatment.

### Melatonin decreases HFD-related inflammation in adipose and liver

**Fig. 7:**
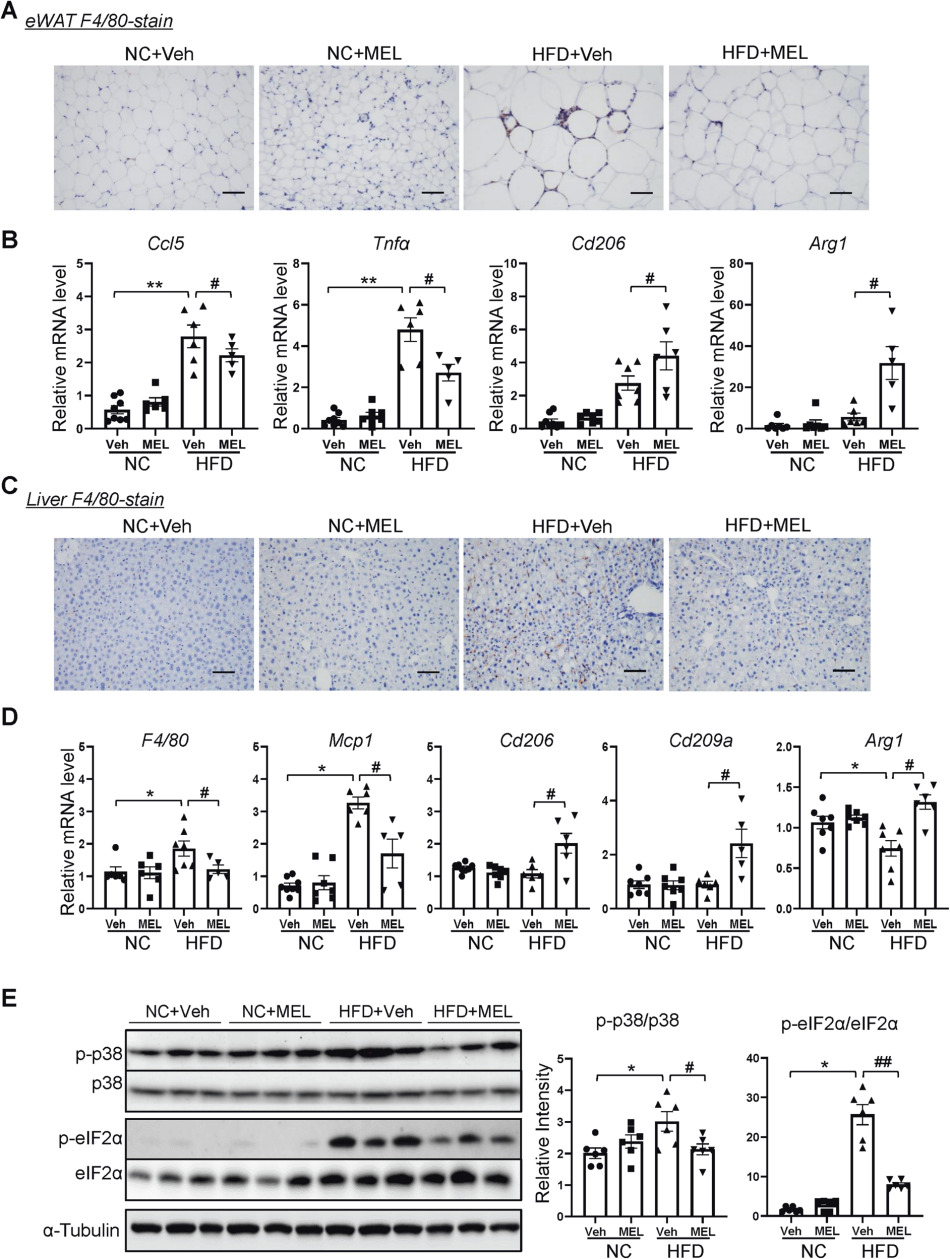
Melatonin attenuates adipose tissue and liver inflammation in HFD-fed mice. **A** F4/80 immunostaining of eWAT. Scale bars = 100 μm. **B** Levels of pro- and anti-inflammatory cytokine mRNAs in eWAT. **C** F4/80 immunostaining of the liver. Scale bars = 100 μm. **D** Levels of F4/80, and pro- and anti-inflammatory cytokine mRNAs, in the liver. **E** Immunoblots of phosphorylated p38 MAPK and eIF2α, and their total protein contents in the liver. Data are mean ± SEM, *n* = 5–8. Significance was determined by one-way ANOVA. *p < 0.05, **p < 0.01 vs. NC + Veh mice; ^#^*p* < 0.05, ^##^*p* < 0.01, vs. HFD + Veh mice.

We assessed the effects of melatonin on adipose inflammation. F4/80 immunostaining revealed marked reductions in macrophage infiltration of hypertrophic adipose and presence of crown-like structures in HFD + MEL mice (Fig. 7A). Proinflammatory cytokines and chemokines (*Ccl5* and *Tnfa)* were associated with decreased eWAT in HFD + MEL mice compared to the HFD mice (Fig. 7B), whereas anti-inflammatory markers (*Cd206* and *Arg1*) increased in HFD + MEL mice (Fig. 7B). Moreover, melatonin resulted in reduced mRNA expression of NADPH oxidase subunits (e.g., *p22*^*phox*^, *p67*^*phox*^, and *Nox4*) in eWAT of HFD mice (Supplementary Fig. 1A). In contrast, melatonin increased eWAT expression of anti-oxidative genes (*Sod*, *Cat*, and *Gpx1*) in DIO mice (Supplementary Fig. 1B).

Similarly, melatonin led to a reduced amount of F4/80^+^ cells (Fig. 7C), decreased gene expression of F4/80 and proinflammatory chemokines, and increased anti-inflammatory marker expression in the liver (Fig. 7D). The decreased hepatic inflammation of melatonin-treated mice was associated with reduced phospho-p38 MAPK and markers of endoplasmic reticulum stress like phospho-eIF2α (Fig. 7E). Additionally, the decrease of hepatic SOD activity caused by the HFD was restored in HFD + MEL mice (Supplementary Fig. 1C). Accordingly, melatonin led to reduced mRNA levels of the NADPH oxidase subunits (Supplementary Fig. 1D) along with higher hepatic expression of anti-oxidative genes in DIO mice (Supplementary Fig. 1E).

### Melatonin no longer protects against obesity in FGF21-deficient mice

**Fig. 8:**
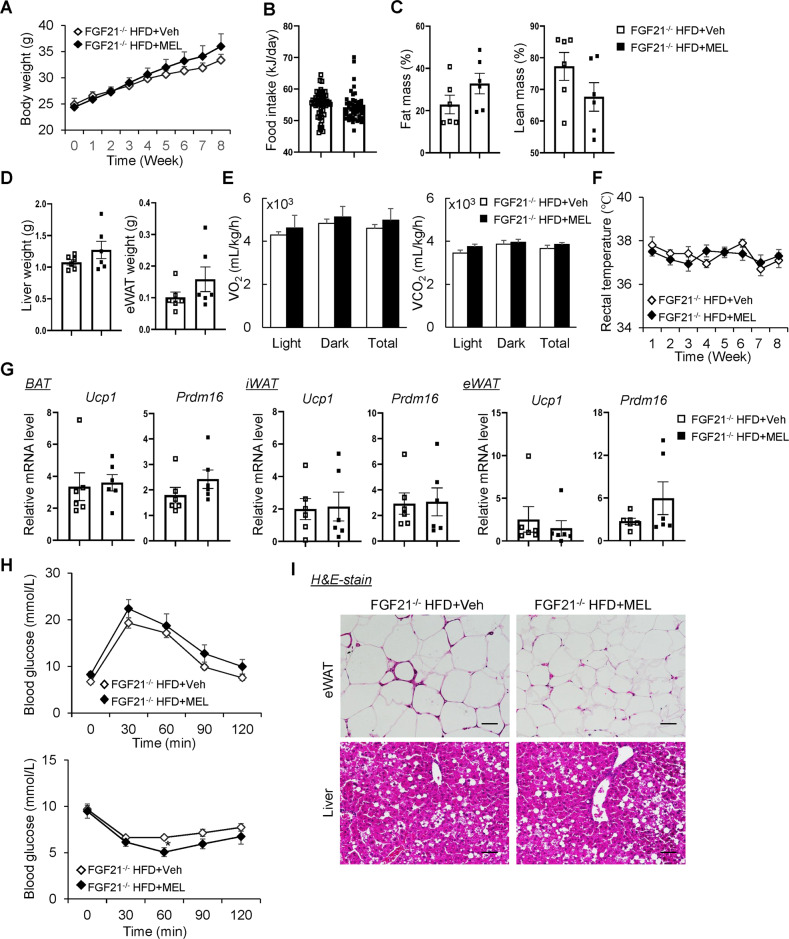
Loss of protection against obesity by melatonin in FGF21^−/−^ mice. **A** Body weight of FGF21^−/−^ mice on a HFD with or without melatonin. **B** Food intake of mice. **C** Fat mass and lean mass of the mice. **D** Tissue weights of the mice. **E** VO_2_ and VCO_2_ of the mice. **F** Rectal temperature of the mice. **G** mRNA levels of thermogenesis-related genes in BAT, iWAT, and eWAT. **H** Glucose and insulin tolerance tests. **I** H&E-stained eWAT and liver sections. Scale bars = 100 μm. Data are mean ± SEM, *n* = 5–6. Significance was determined by one-way ANOVA. *p < 0.05, **p < 0.01 vs. HFD + Veh mice.

FGF21 is a metabolic regulator that responds to stimuli such as cold exposure and HFD by increasing energy expenditure [28]. Notably, FGF21 upregulates UCP1 expression in BAT and beige adipose tissue under cold exposure or obese conditions [16, 29]. Thus, we hypothesized that melatonin suppresses the progression of obesity by increasing UCP1 expression dependent on FGF21. Firstly, to explore the obesogenic role of FGF21, the FGF21^−/−^ mice were fed the HFD or NC for 8 weeks, *ad libitum*. Unexpectedly, a lack of FGF21 reduced body weight of HFD mice, which may have been due to a reduction in fat mass instead of inhibition of food intake (Supplementary Fig. 2A–D). We further investigated whether FGF21 is required for melatonin-mediated attenuation of obesity and insulin resistance by pair-feeding FGF21^−/−^ mice an HFD with or without melatonin for 8 weeks. As expected, melatonin had no effect on body weight, food intake, fat mass, lean mass, or tissue weight (Fig. 8A–D). In addition, melatonin failed to elevate energy expenditure in FGF21^−/−^ mice according to the evaluation of VO_2_ and VCO_2_ (Fig. 8E). Core temperature was not affected by the administration of melatonin in FGF21^−/−^ mice (Fig. 8F). *Ucp1* and *Prdm16* expression was comparable among BAT, iWAT, and eWAT (Fig. 8G). Similarly, melatonin did not mediate HFD-induced glucose intolerance or insulin resistance in FGF21^−/−^ mice (Fig. 8H). Lastly, adiposity and fatty liver were similar between the wild-type and FGF21^−/−^ mice (Fig. 8I). Therefore, melatonin prevented obesity and insulin resistance dependent on FGF21 expression.

## Discussion

Excessive energy intake and/or insufficient energy utilization contribute to obesity. Increasing energy expenditure offers one potential therapeutic approach for obesity and metabolic syndrome. In the present study, we provide solid evidence that melatonin regulated energy homeostasis and ameliorated insulin resistance and inflammation caused by obesity. We showed that treating HFD-fed mice with melatonin reduced weight gain, adiposity, and ectopic fat accumulation despite pair-feeding conditions. Furthermore, melatonin shifted energy metabolism toward fat usage, accompanied by enhanced skeletal muscle AMPKα phosphorylation and increased levels of FGF21. Melatonin also enhanced energy expenditure, heat production, and UCP1 in BAT, suggesting that melatonin promoted BAT activity. Notably, melatonin did not affect energy expenditure in NC mice, nor did it affect body weight or insulin resistance, suggesting that melatonin treatment had no toxicity and/or side effects on normal mice in an energy-balanced condition. The apparent protective effects of melatonin were not observed in FGF21-deficient mice, which suggests that melatonin may attenuate obesity through an FGF21-dependent mechanism.

Melatonin is a hormone known to reset the internal circadian clock in mammals, and is also secreted rhythmically. It has been demonstrated that administering melatonin before the lights off regulate the circadian rhythm imbalance [30] and attenuate the progression of obesity in HFD mice [31]. We therefore treat the melatonin before dark with peritoneal injection. Melatonin increases food intake in rodents [32, 33]. Our preliminary study also confirmed that melatonin increased food intake among HFD mice, but did not influence body weight (data not shown). Importantly, we pair-fed the mice in order to avoid increased food intake and better isolate the effects of melatonin.

BAT functions to generate heat and maintain body temperature by uncoupling oxidative phosphorylation from ATP synthesis [34]. More recently, BAT has been shown to combat obesity and promote glucose and lipid homeostasis in humans and rodents. Melatonin increases BAT activity in Djungarian hamsters [35] and this finding has been supported by subsequent studies [36, 37]. Melatonin thereby modulates energy homeostasis and protects against obesity and its associated syndromes [15, 37, 38]. In this study, melatonin-treated mice consumed more oxygen and produced more CO_2_, leading to overall increased energy expenditure, and they had increased UCP1 in BAT. These results suggest that the melatonin treatment increased BAT-mediated thermogenic activity, and subsequently regulated energy metabolism and combated obesity.

FGF21 plays a regulatory role in lipolysis in WAT [39], increases hepatic fatty acid oxidation [40], and regulates the differentiation to brown adipocytes. FGF21 induces upregulation of local PGC-1α, thus promoting thermogenesis in BAT and skeletal muscle [18]. In addition, FGF21 mediates cold-induced thermogenesis through BAT activation in humans [41]. Lucas et al. demonstrated that FGF21 signals directly modulate brown adipocyte activity and subsequently increase insulin sensitivity [42]. FGF21 activates β3-adrenergic receptors in regulates browning of WAT [18]. FGF21 administration promotes elevated energy expenditure and, consequently, weight loss in mice [28]. Thus, the increase FGF21 in plasma and tissues following melatonin contribute to fat utilization and BAT activity in obesity. Importantly, melatonin did protect against inflammation or insulin resistance in FGF21^−/−^ mice. However, the mechanism of melatonin-induced FGF21 has not been clarified in the present study and warrants further investigation.

Consistent with previous studies [6, 8, 11], melatonin attenuated weight gain and development of fatty liver induced by an HFD. Our findings suggest that increased energy expenditure and oxidation of fatty acids play mechanistic roles in weight reduction. Melatonin enhanced AMPKα phosphorylation in skeletal muscle which, once activated, restores energy homeostasis through the activation of catabolic pathways (e.g., oxidation of fatty acids) and inhibition of anabolic pathways (e.g., synthesis of fatty acids). In this manner, increased activation of AMPK may drive melatonin’s anti-obesity effects.

AMPK signaling generates a similar metabolic profile to that of FGF21 [43]. Endocrine signaling of FGF21 activates the AMPK pathway, which in turn activates local AMPK signaling [43] and promotes fatty acid oxidation and lipolytic action. Here, we demonstrated that melatonin increased hepatic FGF21 and enhanced AMPK activation in skeletal muscle, resulting in elevated fatty acid oxidation and utilization.

Melatonin is well known as an antioxidant agent. Here, we showed that the improvement of obesity observed in the HFD + MEL mice was accompanied by reduced oxidative stress in eWAT and liver [44]. Reactive oxygen species (ROS) production and the secretion of inflammatory cytokines like TNFα and C-C motif chemokine ligand 2 are elevated under conditions of excess nutrition, which eventually impairs insulin signaling and results in insulin resistance [45]. In this study, melatonin reduced mRNA expression of NADPH subunits, which contribute to adipocyte and hepatocyte ROS production, whereas melatonin enhanced hepatic SOD activity and increased antioxidant enzyme expression in eWAT and the liver. These findings support the conclusion that melatonin exerts anti-oxidative effects and protects against obesity-related insulin resistance.

In conclusion, melatonin aids in the prevention of obesity and controlling energy metabolism by increasing energy expenditure and fat utilization. Melatonin-mediated antioxidant activity further improved insulin resistance and hepatic steatosis in diet-related obesity. Thus, melatonin is a promising therapeutic agent in treating obesity and its comorbidities, including diabetes and fatty liver disease.

## Supplementary information

Supplementary tables and figures

## Data Availability

All data generated and analyzed during this study are included in this published article and its supplementary information files.
