# Supplementary material for: Suppression of obesity by melatonin through increasing energy expenditure and accelerating lipolysis in mice fed a high-fat diet
Source: Nutr Diabetes. 2022 Oct 7;12:42. doi: 10.1038/s41387-022-00222-2 (PMC9546869; doi:10.1038/s41387-022-00222-2)
Supplement: Supplementary file 1 — Supplementary tables and figures [file 41387_2022_222_MOESM1_ESM.docx]

**Supplementary Tables:**

**Supplementary Table 1–Composition of the normal chow (NC, D12450B) and high-fat diet (HFD, D12492)**

| **Ingredient** | **NC (wt%)** | **HFD (wt%)** |
| --- | --- | --- |
| Casein, Lactic, 30 Mesh | 19.0 | 25.8 |
| Cystine, L | 0.3 | 0.4 |
| Sucrose, Fine Granulated | 33.6 | 9.4 |
| Starch, Corn | 29.9 | 0 |
| Lodex 10 | 3.3 | 16.2 |
| Solka Floc, FCC200 | 4.7 | 6.5 |
| Soybean Oil, USP | 2.4 | 3.2 |
| Lard | 1.9 | 31.7 |
| S10026B | 4.7 | 6.5 |
| Choline Bitartrate | 0.2 | 0.3 |
| V10001C | 0.1 | 0.1 |

| **Nutrition facts** | **NC (Kcal%)** | **HFD (Kcal%)** |
| --- | --- | --- |
| Protein | 20 | 20 |
| Carbohydrate | 70 | 20 |
| Fat | 10 | 60 |

**Supplementary Table 2–Mice primer sequences.**

| Gene | 5’ Primer | 3’ Primer |
| --- | --- | --- |
| mAcox1 | TTATGCGCAGACAGAGATGG | AGGCATGTAACCCGTAGCAC |
| mArg1 | CTCCAAGCCAAAGTCCTTAGAG | AGGAGCTGTCATTAGGGACATC |
| mCat | CCAGCGACCAGATGAAGCAG | CCACTCTCTCAGGAATCCGC |
| mCcl5 | TGCCCTCACCATCATCCTCACT | GGCGGTTCCTTCGAGTGACA |
| mCd206 | CAAGGAAGGTTGGCATTTGT | CCTTTCAGTCCTTTGCAAGC |
| mCidea | ATCACAACTGGCCTGGTTACG | TACTACCCGGTGTCCATTTCT |
| mCpt1α | AAACCCACCAGGCTACAGTG | TCCTTGTAATGTGCGAGCTG |
| mElovl3 | TCCGCGTTCTCATGTAGGTCT | GGACCTGATGCAACCCTATGA |
| mF4/80 | CTTTGGCTATGGGCTTCCAGTC | GCAAGGAGGACAGAGTTTATCGTG |
| mFas | AGAGACGTGTCACTCCTGGACTT | GCTGCGGAAACTTCAGAAAAT |
| mFgf21 | GTGTCAAAGCCTCTAGGTTTCTT | GGTACACATTGTAACCGTCCTC |
| mCd209a | CCTGGGAGAGGAAGACTGTG | CTTGCTAGGGCAGGAAGTTG |
| mCpt1α | AAACCCACCAGGCTACAGTG | TCCTTGTAATGTGCGAGCTG |
| mNox4 | TTGGGTCAGCACTGGCTCTG | TGGCGGTGTGCAGTGCTATC |
| mGpx1 | TTCGGACACCAGGAGAATGG | TAAAGAGCGGGTGAGCCTTC |
| mMcp1 | AGGTCCCTGTCATGCTTCTGG | CTGCTGCTGGTGATCCTCTTG |
| mLcad | TCACCACACAGAATGGGAGA | ACGCTTGCTCTTCCCAAGTA |
| mP^22phox^ | GTCCACCATGGAGCGATGTG | CAATGGCCAAGCAGACGGTC |
| mP^67phox^ | CTGGCTGAGGCCATCAGACT | AGGCCACTGCAGAGTGCTTG |
| mPgc-1α | ATGTGTCGCCTTCTTGCTCT | ATCTACTGCCTGGGGACCTT |
| mPparα | GAGGGTTGAGCTCAGTCA GG | GGTCACCTACGAGTGGCATT |
| mPrdm16 | GGCGAGGAAGCTAGCCAAA | GGTCTCCTCCTCGGCACTCT |
| mScd | CATCATTCTCATGGTCCTGCT | CCCAGTCGTACACGTCATTTT |
| mSod | CAGCATGGGTTCCACGTCCA | CACATTGGCCACACCGTCCT |
| mSrebp-1c | GGAGCCATGGATTGCACATT | GGCCCGGGAAGTCACTGT |
| mTnfα | AAGCCTGTAGCCCACGTCGTA | GGCACCACTAGTTGGTTGTCTTTG |
| mUcp1 | ACTGCCACACCTCCAGTCATT | CTTTGCCTCACTCAGGATTGG |
| mβ-actin | AGGCCCAGAGCAAGAGAGGTA | GGGGTGTTGAAGGTCTCAAACA |
| m18S | AGG CCC AGA GCA AGA GAG GTA | GGG GTG TTG AAG GTC TCA AAC A |

**Supplementary Table 3–Antibodies used in immunoblotting.**

| Antibody |  |
| --- | --- |
| anti-phospho-AMPK α | Cell signaling (#2535) |
| anti-AMPK α | Cell signaling (#2532) |
| anti-phospho-p38 MAPK | Cell signaling (#9211) |
| anti-p38 MAPK | Cell signaling (#9212) |
| anti-phospho-Akt | Cell signaling (#9271) |
| anti-Akt | Cell signaling (#9272) |
| anti-phospho-eIF2α | Cell signaling (#3597) |
| anti-eIF2α | Cell signaling (#9722) |
| anti-FGF21 | Abcam (ab171941) |
| anti-UCP1 | Abcam (ab10983) |
| anti-α-Tubulin | Cell signaling (#2144) |

**Supplementary Figures**

**
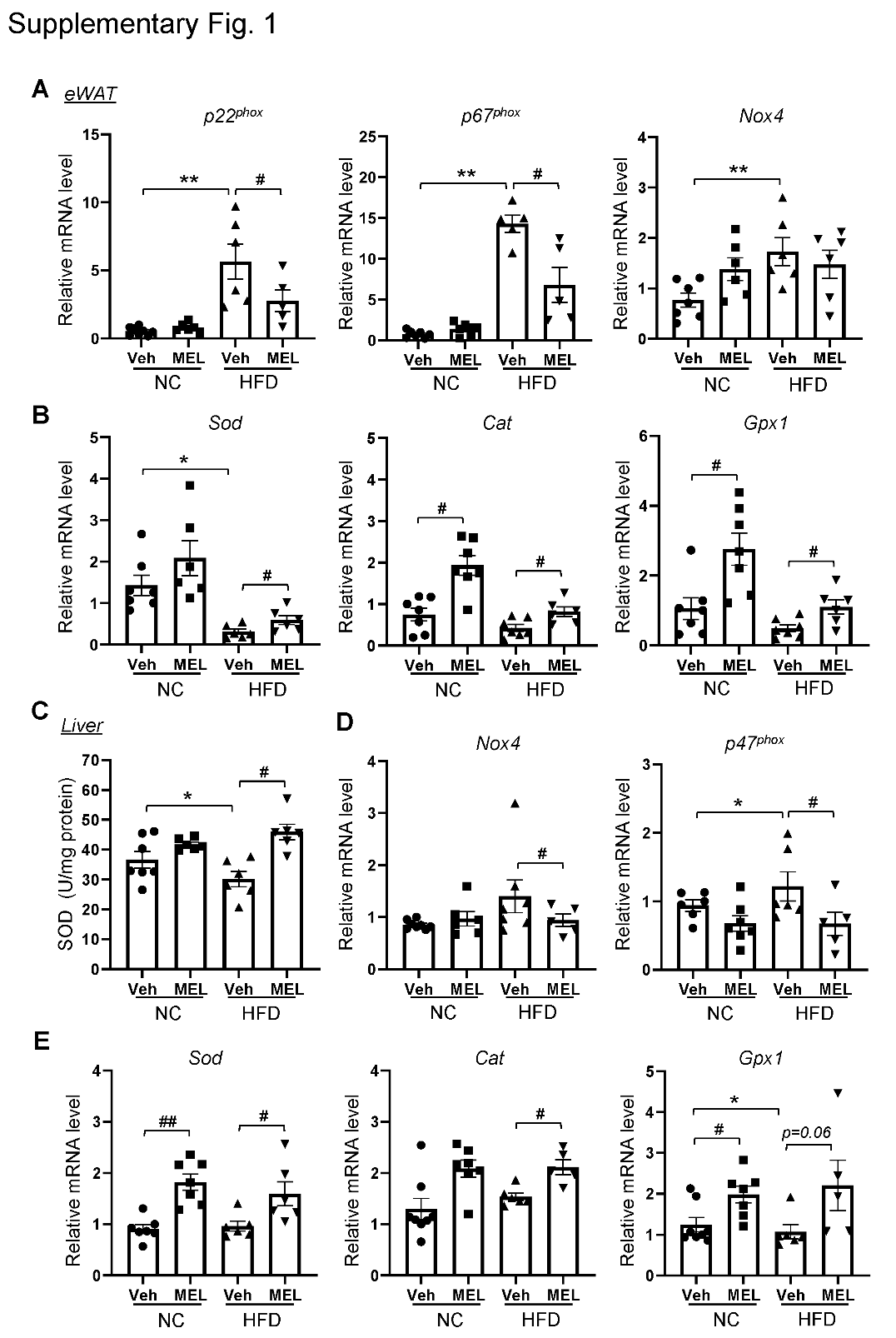
**

**Supplementary Fig. 1 Melatonin attenuates oxidative stress in the eWAT and liver of DIO mice.** (A) mRNA levels of NADPH oxidase genes in eWAT. (B) mRNA levels of anti-oxidative stress genes in eWAT. (C) SOD activity in liver. (D) mRNA levels of NADPH oxidase genes in liver. (E) mRNA levels of anti-oxidative stress-related genes in liver. Data are mean ± SEM, n = 5–8. Significance was determined by one-way ANOVA. **p* < 0.05, ***p* < 0.01 vs. NC + Veh mice; #*p* < 0.05, ##*p* < 0.01, vs. HFD + Veh mice.


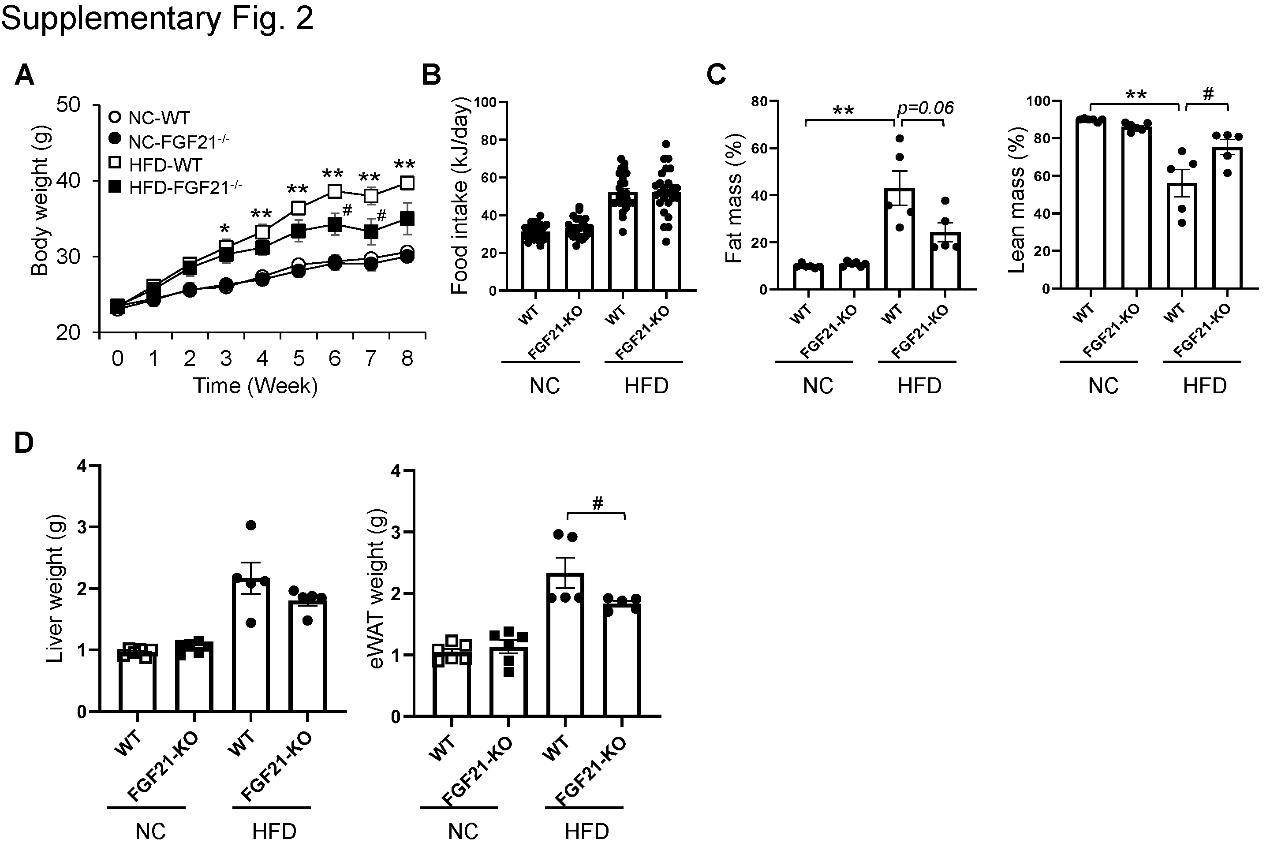


**Supplementary Fig. 2 Fgf21 deficiency decreases body weight and fat mass in HFD-fed mice.** (A) Body weight of mice. (B) Energy intake of mice. (C) Body composition of mice. (D) Tissue weight of mice. Data are mean ± SEM, n = 5–6. Significance was determined by one-way ANOVA. **p* < 0.05, ***p* < 0.01 vs. HFD mice; *^#^p* < 0.05, *^##^p* < 0.01, vs. wild type mice.
